# Probing the Catalytic Mechanism of *Vibrio harveyi* GH20 *β-N*-Acetylglucosaminidase by Chemical Rescue

**DOI:** 10.1371/journal.pone.0149228

**Published:** 2016-02-12

**Authors:** Piyanat Meekrathok, Wipa Suginta

**Affiliations:** 1 Biochemistry-Electrochemistry Research Unit and School of Biochemistry, Institute of Science, Suranaree University of Technology, Nakhon Ratchasima, 30000, Thailand; 2 Center of Excellence in Advanced Functional Materials, Suranaree University of Technology, Nakhon Ratchasima, 30000, Thailand; Russian Academy of Sciences, Institute for Biological Instrumentation, RUSSIAN FEDERATION

## Abstract

**Background:**

*Vibrio harveyi* GH20 *β*-*N*-acetylglucosaminidase (*Vh*GlcNAcase) is a chitinolytic enzyme responsible for the successive degradation of chitin fragments to GlcNAc monomers, activating the onset of the chitin catabolic cascade in marine *Vibrios*.

**Methods:**

Two invariant acidic pairs (Asp303-Asp304 and Asp437-Glu438) of *Vh*GlcNAcase were mutated using a site-directed mutagenesis strategy. The effects of these mutations were examined and the catalytic roles of these active-site residues were elucidated using a chemical rescue approach. Enhancement of the enzymic activity of the *Vh*GlcNAcase mutants was evaluated by a colorimetric assay using *p*NP-GlcNAc as substrate.

**Results:**

Substitution of Asp303, Asp304, Asp437 or Glu438 with Ala/Asn/Gln produced a dramatic loss of the GlcNAcase activity. However, the activity of the inactive D437A mutant was recovered in the presence of sodium formate. Our kinetic data suggest that formate ion plays a nucleophilic role by mimicking the β-COO^-^side chain of Asp437, thereby stabilizing the reaction intermediate during both the glycosylation and the deglycosylation steps.

**Conclusions:**

Chemical rescue of the inactive D437A mutant of *Vh*GlcNAcase by an added nucleophile helped to identify Asp437 as the catalytic nucleophile/base, and hence its acidic partner Glu438 as the catalytic proton donor/acceptor.

**General Significance:**

Identification of the catalytic nucleophile of *Vh*GlcNAcases supports the proposal of a substrate-assisted mechanism of GH20 GlcNAcases, requiring the catalytic pair Asp437-Glu438 for catalysis. The results suggest the mechanistic basis of the participation of *β*-*N*-acetylglucosaminidase in the chitin catabolic pathway of marine *Vibrios*.

## Introduction

*Vibrio harveyi* is a bioluminescent marine bacterium that utilizes chitin biomaterials, which are abundantly available in the aquatic environment, as its sole source of energy. The initial step of chitin breakdown by *Vibrios* involves the synergistic action of various chitin-related proteins [1–3]. Lytic polysaccharide monooxygenase, a copper-dependent enzyme, attacks recalcitrant chitin polysaccharides [4,5], while endochitinases hydrolyse long chitin chains to chitin oligosaccharides, which are then transported through the bacterial cell wall by chitoporin or ChiP [6–8]. In the periplasm, these chitin fragments are degraded by exo *β*-*N*-acetylglucosaminidases (GlcNAcases) and the resultant GlcNAc monomers are transported through the inner membrane by the GlcNAc-PTS transporter and finally metabolized in the cytoplasm, finally acting as sources of carbon and nitrogen [9]. Based on CAZy (Carbohydrate-Active enZYmes Database; http://www.cazy.org), bacterial GlcNAcases belong to either glycoside hydrolase family 3 (GH3), family 20 (GH20) or family 84 (GH84). In general, GlcNAcases from all families break the *β*-1,4-glycosidic linkage next to the non-reducing end of GlcNAc-containing oligosaccharides, generating GlcNAc units as the end product [10,11]. However, GH3 GlcNAcases differ in their amino acid sequence identity and mode of action from those in the GH20 and GH84 families [12,13]. GH3 GlcNAcases catalyse the hydrolytic reaction through a standard ‘retaining’ mechanism involving a covalent glycosyl-enzyme intermediate [14,15], while GH20 and GH84 GlcNAcases hydrolyse chitooligosaccharides through a ‘substrate-assisted’ mechanism involving the transient formation of an oxazolinium ion intermediate [16–18].

A ‘chemical rescue’ approach has been employed to identify the catalytic mechanism of several glycoside hydrolases. The effects of exogenous anions, such as azide or formate, can provide direct evidence identifying the catalytic acid/base residues in retaining glycoside hydrolases. Following mutation of the acid-base residue or the nucleophilic residue, hydrolytic activity of the mutants can be rescued by the addition of an exogenous nucleophile, such as azide ion, resulting in the formation of products with the α or β configuration. An example is a study on *Bacillus* 1,3–1,4-*β*-D-glucan 4-glucanohydrolases [19]. Sodium azide was shown to rescue the glucanase activity, but with a different mechanism, when either the nucleophilic (Glu134) or the catalytic acid/base (Glu138) residues were mutated to Ala. E138A yielded a β-glycosyl azide product, arising from nucleophilic attack of azide on the glycosyl-enzyme intermediate, thus proving the role Glu138 as the catalytic acid-base residue. In contrast, azide reactivated the E134A mutant through a single inverting displacement to give the α-glycosyl azide product, consistent with Glu134 being the catalytic nucleophile.

In the substrate-assisted mechanism of GH20 enzymes, chemical rescue helps to directly identify the catalytic nucleophile in the catalytic pair (typically the invariant Asp-Glu couple) in the enzyme’s active site. The Asp residue normally acts as the catalytic base/nucleophile, while the glutamic acid acts as the catalytic proton donor/acceptor [20,21]. Examples of enzymes studied by use of this approach include *Streptomyces plicatus* GH20 hexoxaminidase (*Sp*Hex) [21], *Arthrobactor protophormiae* GH85 endo-*β*-*N*-acetylglucosaminidase (Endo A) [22], *Streptomyces* sp. GH1 *β*-glucosidase [23], *Paenibacillus* sp. TS12 GH3 glucosylceraminidase [24], *Cellulomonas fimi* GH10 exoglucanase/xylanase [25], *Bacillus licheniformis* GH16 1,3–1,4-*β*-glucanase [19], *Sulfolobus solfataricus* GH29 *α*-L-fucosidase [26] and *Geobacillus stearothermophilus* T-6 GH51 *α*-L-arabinofuranosidase [27]. In the case of GH20 GlcNAcases, rescue of the activity of *Sp*Hex from *Streptomyces plicatus* [21] has been demonstrated. *Sp*Hex catalyses the hydrolysis of *N*-acetyl-*β*-hexosaminides. Point mutation of Asp313 of *Sp*Hex to Ala or Asn (mutants D313A or D313N) almost abolished the enzyme’s hydrolytic activity, but the catalytic activity of the mutant D313A was significantly increased with the inclusion of sodium azide in the assay medium.

We previously cloned, expressed and characterized a novel member of the GH20 GlcNAcase family, from the marine bacterium *V*. *harveyi* (so-called *Vh*GlcNAcase) [9]. Based on amino acid sequence alignment with other GlcNAcases, the catalytic pair of *Vh*GlcNAcase was predicted to be Asp437-Glu438. We have now employed the chemical rescue approach to identify the functional roles of Asp437 as the catalytic nucleophile and Glu438 as the catalytic acidic residue of *Vh*GlcNAcase.

## Materials and Methods

### Bacterial strains and chemicals

*Escherichia coli* type strain *DH5α* was used for cloning, subcloning and plasmid preparation. Supercompetent *E*. *coli* XL1Blue (Stratagene, La Jolla, CA, USA) was the host strain for the production of mutagenized plasmid. *E*. *coli* strain M15 (pREP) host cells (Qiagen, Valencia, CA, USA) and the recombinant plasmid of pQE 60 vector containing *GlcNAcase* gene fragments were used for high-level expression of recombinant enzyme. Chemicals and reagents used for protein expression, purification and characterization of *Vh*GlcNAcase were of analytical grade unless otherwise stated. A QuickChange Site-Directed Mutagenesis Kit including *Pfu* Turbo DNA polymerase was purchased from Stratagene. Restriction enzymes and DNA modifying enzymes were the products of New England Biolabs, Inc. (Beverly, MA, USA). All other chemicals and reagents were obtained from the following sources: reagents for bacterial media (Scharlau Chemie S.A., Barcelona, Spain); *p*-nitrophenol (*p*NP) and *p*-nitrophenyl-*N*-acetyl-glucosaminide (*p*NP-GlcNAc) were purchased from Sigma-Aldrich (St. Louis, MO, USA); sodium azide was purchased from LabChem Inc. (Zelienople, PA, USA); sodium nitrate, sodium formate and sodium chloride were purchased from Carlo Erba (Rodano, Milano, Italy).

### Amino acid sequence analysis and homology modeling

The amino acid sequence of the matured *Vh*GlcNAcase was submitted to Swiss-Model (http://swissmodel.expasy.org/) for tertiary structure prediction using the crystal structure of *S*. *marcescens* chitobiase (PDB entry: 1QBA) as a structural template. To obtain detailed information about the enzyme’s active site, the modelled structure of *Vh*GlcNAcase was superimposed on the 3D structure of *S*. *marcescens* chitobiase (*Sm*CHB) docked with diNAG coordinates. The annotated structures were edited and displayed in PyMOL (www.pymol.org). The structure-based alignment was generated by aligning the amino acid sequence of *Vh*GlcNAcase with five GH20 GlcNAcases with known 3D-structures, including *S*. *marcescens* chitobiase, *Sm*CHB (PDB code: 1QBA); *Streptomyces plicatus β*-*N*-acetylhexosaminidase, *Sp*Hex (PDB code: 1HP4); *Paenibacillus* sp. *β*-hexosaminidase, *Ps*Hex1T (PDB code: 3GH4); human *β*-hexosaminidase A (*α*-chain), *Hs*HexA (PDB code: 2GJX) and human *β*-hexosaminidase B (*β*-chain), *Hs*HexB (PDB code: 1NOU). The amino acid sequence alignment was carried out in ClustalW, and the structure-based alignment was further generated using the program ESPript, v3.0 [28].

### Site-directed mutagenesis

The pQE 60 expression vector harboring the full length *VhGlcNAcase* cDNA [9] was used as DNA template. Site-directed mutagenesis was carried out using the QuickChange Site-Directed Mutagenesis Kit (Stratagene), following the Manufacturer’s instruction. The mutagenic primers were synthesized by commercial sources (BioDesign Co., Ltd Bangkok, Thailand and Bio Basic Canada Inc., Ontario, Canada) and the oligonucleotide sequences of these primers are listed in Table 1. Eight single mutants, namely D303A, D303N, D304A, D304N, D437A, D437N, E438A and E438Q, were generated and the success of the designed mutations was verified by automated DNA sequencing (First BASE Laboratories Sdn Bhd, Selangor Darul Ehsan, Malaysia).

**Table 1:** Primers used for site-directed mutagenesis.

Mutation	Oligonucleotide sequence^a^
D303A	forward 5′- CATTGGCATCTCACTGCGGATGAAGGCTGGCGTG -3′
	reverse 5′- CACGCCAGCCTTCATCCGCAGTGAGATGCCAATG -3′
D303N	forward 5′- CATTGGCATCTCACTAACGATGAAGGCTGGCGTG -3′
	reverse 5′- CACGCCAGCCTTCATCGTTAGTGAGATGCCAATG -3′
D304A	forward 5′- GCATCTCACTGACGCGGAAGGCTGGCGTGTC -3′
	reverse 5′- GACACGCCAGCCTTCCGCGTCAGTGAGATGC -3′
D304N	forward 5′- GCATCTCACTGACAACGAAGGCTGGCGTGTC -3′
	reverse 5′- GACACGCCAGCCTTCGTTGTCAGTGAGATGC -3′
D437A	forward 5′- GTTCACATTGGCGCGGCGGAAGTGCCTAACGGC -3′
	reverse 5′- GCCGTTAGGCACTTCCGCCGCGCCAATGTGAAC -3′
D437N	forward 5′- GTTCACATTGGCGCGAACGAAGTGCCTAACGGC -3′
	reverse 5′- GCCGTTAGGCACTTCGTTCGCGCCAATGTGAAC -3′
E438A	forward 5′- CACATTGGCGCGGACGCGGTGCCTAACGGCGTGTG -3′
	reverse 5′- CACACGCCGTTAGGCACCGCGTCCGCGCCAATGTG -3′
E438Q	forward 5′- GTTCACATTGGCGCGGACCAGGTGCCTAACGGCGTGTG -3′
	reverse 5′- CACACGCCGTTAGGCACCTGGTCCGCGCCAATGTGAAC -3′

^a^ Sequences underlined indicate mutated codons.

### Protein expression and purification

The recombinant wild-type *Vh*GlcNAcase was expressed in *E*. *coli* M15 (pREP) cells as a 652-amino acid polypeptide, including the *C*-terminal (His)_6_ sequence [9]. Expression of all GlcNAcase variants was based on the protocol described previously by Suginta *et al*. [9]. Briefly, the transformed cells were grown at 37°C in Terrific Broth (TB) containing 100 μg mL^-1^ ampicillin and 25 μg mL^-1^ kanamycin until the cell density reached an OD_600_ of 0.6. The cell culture was cooled to 20°C, before isopropyl thio-*β*-D-galactoside (IPTG) was added to a final concentration of 0.4 mM for GlcNAcase expression. Cell growth was continued at 20°C for an additional 18 h, and cells were harvested by centrifugation at 4,500 ×*g* for 30 min. The bacterial pellet was re-suspended in lysis buffer (20 mM Tris-HCl buffer, pH 8.0, 150 mM NaCl, 1 mM phenylmethylsulphonyl fluoride (PMSF), 5% (v/v) glycerol and 1 mg mL^-1^ lysozyme, and then lysed on ice using a Sonopuls ultrasonic homogenizer with a 6-mm diameter probe (50% duty cycle; amplitude setting, 30%; total time, 30 s, 6–8 times). Unbroken cells and cell debris were removed by centrifugation at 12,000 ×*g* for 1 h. The supernatant containing *Vh*GlcNAcase was immediately applied to a polypyrene column packed with 5 mL of TALON^®^ Superflow^™^ metal affinity resin (Clontech Laboratories, Inc., USA) operated at 4°C with gravity-dependent flow. The column was washed with 8 column volumes (cv) of equilibration buffer (20 mM Tris-HCl buffer, pH 8.0 containing 150 mM NaCl), followed by 7 cv of the equilibration buffer containing 10 mM imidazole. The protein was then eluted with 250 mM imidazole in the same buffer. Eluted fractions of 10 mL were collected and 15 μL of each fraction was analyzed by 12% SDS-PAGE, according to the method of Laemmli [29], to confirm the purity of the protein. Fractions with GlcNAcase activity were pooled and subjected to several rounds of centrifugation in Vivaspin-20 ultrafiltration membrane concentrators (10 kDa molecular-weight cut-off, Vivascience AG, Hannover, Germany) for complete removal of imidazole. The final concentration of the protein was determined by the BCA method [30]. The freshly prepared protein was either immediately used for functional characterization or stored at -80°C until used.

### GlcNAcase activity assay

GlcNAcase activity was determined by a colorimetric assay using *p*NP-GlcNAc as substrate. The reaction mixture in a 96-well microtiter plate contained an optimal amount of *Vh*GlcNAcase (0.1 μg for WT and 5 μg for mutants), 500 μM *p*NP-GlcNAc and 100 mM phosphate buffer, pH 7.0 in a total volume of 200 μL. When sodium formate was added, the final pH in the reaction mixture was found to be 7.0 ± 0.3. The assay was carried out at 37°C with constant agitation in an Eppendorf ThermoMixer^®^ Comfort (Eppendorf AG, Hamburg, Germany), and was terminated by adding 100 μL of 3 M Na_2_CO_3_ to each well after 10 min. The concentration of *p*-nitrophenol (*p*NP) released was determined at 405 nm in a Biochrom Anthos MultiRead 400 Microplate Reader (Biochrom, Cambridge, UK). The molar quantity of the liberated *p*NP was calculated from a calibration curve with *p*NP concentration varied from 0 to 20 nmol. The hydrolytic activity of the enzyme was expressed as the quantity of *p*NP (nmol) produced in 1 min at 37°C.

### Determination of the pH optima of *Vh*GlcNAcase WT and D437A mutant

To obtain the activity/pH profiles, the specific activity of *Vh*GlcNAcase WT and D437A mutant was determined in a discontinuous assay. The reaction mixture contained 0.05 μg *Vh*GlcNAcase or 5 μg D437A, 500 μM *p*NP-GlcNAc, and McIlvaine’s sodium phosphate-citric acid buffer, pH 3.0–9.0 [31] at different pH values ranging from 3.0 to 9.0, in a total volume of 200 μL. The reaction was carried out as described for the GlcNAcase activity assay.

### Chemical rescue assay

Sodium azide and sodium formate were initially tested for their ability to rescue the enzymic activity of inactive *Vh*GlcNAcase mutants. A 200-μL assay mixture, prepared in a 96-well microtiter plate, contained 500 μM *p*NP-GlcNAc, 5 μg of enzyme, 1 M sodium azide or formate and 100 mM sodium phosphate buffer, pH 7.0. The reaction mixture was incubated at 37°C for 10 min with constant agitation, and the reaction was terminated by the addition of 100 μL of 3 M Na_2_CO_3_. The reaction of wild-type *Vh*GlcNAcase was carried out as described for mutants, but with 0.1 μg of the enzyme in the assay.

To determine the effect of concentrations on the rescued activity, the reaction mixture was incubated with different concentrations of azide or formate for a longer time. A 200-μL assay mixture contained 500 μM *p*NP-GlcNAc, 0.2 μg of mutants and 0.1–2.0 M sodium azide or formate in 100 mM sodium phosphate buffer, pH 7.0. The reaction was allowed to proceed at 37°C for 60 min, and then terminated by the addition of 100 μL of 3 M Na_2_CO_3_. The amount of *p*NP released was calculated as described above.

### Time-course and kinetics of sodium formate effects on the activity of *Vh*GlcNAcase D437A mutant

Chemical rescue of the inactive D437A mutant by sodium formate was further observed at different times of incubation. A 200-μL assay mixture contained 500 μM *p*NP-GlcNAc, 0.2 μg of the mutant D437A and 0.1–2.0 M sodium formate in 100 mM sodium phosphate, pH 7.0. The reaction mixture was incubated at 37°C for times of 0, 2.5, 5, 10, 30, and 60 min. For kinetic experiments, a 200-μL reaction mixture, containing 0–500 μM *p*NP-GlcNAc, 5 μg of the mutant D437A, 0.1–2.0 M sodium formate and 100 mM sodium phosphate buffer, pH 7.0, was incubated for 10 min at 37°C and the reaction terminated with 100 μL of 3 M Na_2_CO_3_. The amount of the *p*NP formed during the reaction was estimated as described previously. The kinetic parameters (apparent *K*_m_, apparent *k*_cat_ and apparent *k*_cat_/*K*_m_) were determined with a non-linear regression function available in GraphPad Prism v.5.0 (GraphPad Software Inc., San Diego, CA).

## Results

### Sequence analysis and homology modeling

**Fig 1:**
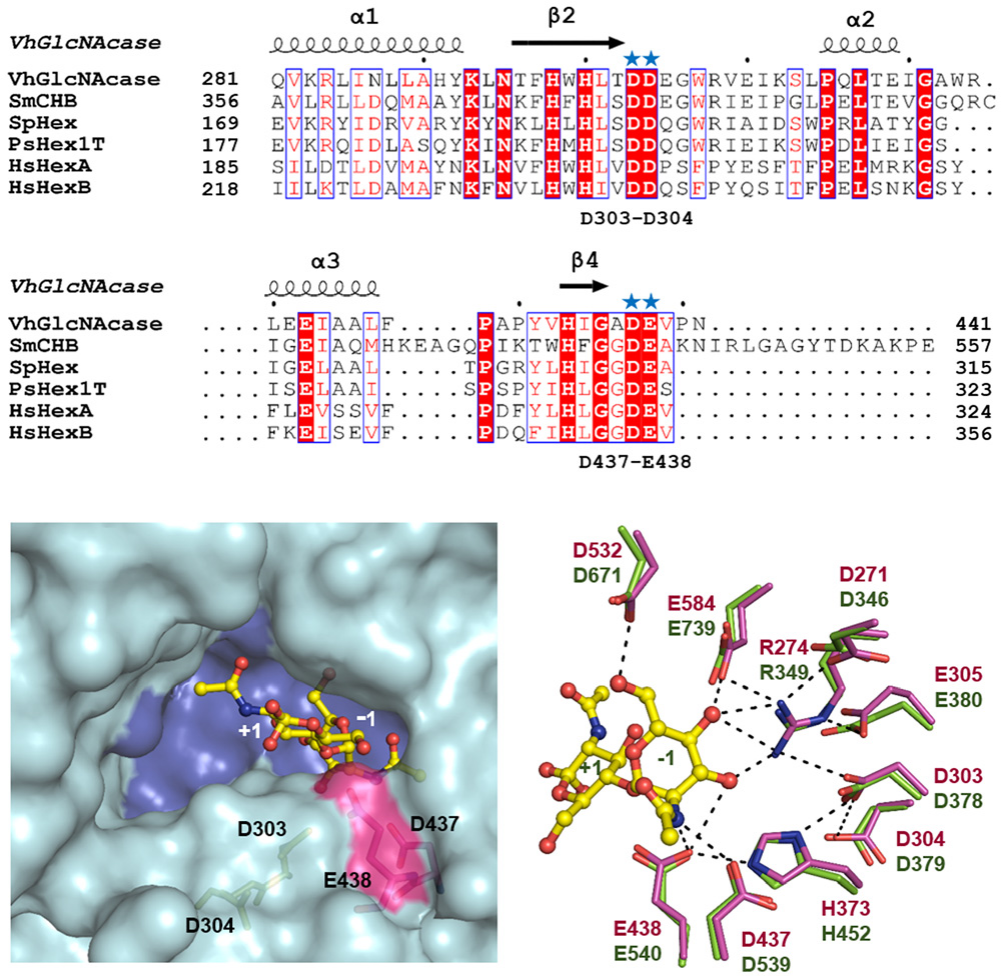
Comparison of the modelled structure of *Vh*GlcNAcase with other GlcNAcases. (A) Multiple sequence alignment of GH20 glycoside hydrolases. The amino acid sequence of *Vibrio harveyi β*-*N*-acetylglucosaminidase, *Vh*GlcNAcase (SwissProt: D9ISE0) was retrieved from the Uniprot database. This sequence was aligned with those of *Serratia marcescens* chitobiase, *Sm*CHB (SwissProt: Q54468), *Streptomyces plicatus β*-*N*-acetylhexosaminidase, *Sp*Hex (SwissProt: O85361), *Paenibacillus* sp. *β*-hexosaminidase, *Ps*Hex1T (SwissProt: D2KW09), human *β*-hexosaminidase A (*α*-chain), *Hs*HexA (SwissProt: P06865) and human *β*-hexosaminidase B (*β*-chain), *Hs*HexB (SwissProt: P07686). The putative amino acid residues that are important for GlcNAcase activity are indicated with blue stars. (B) Surface representation of the active-site pocket of *Vh*GlcNAcase (in blue) docked with GlcNAc_2_ (in yellow stick) from *Sm*CHB (PDB entry: 1QBB). The solvent-accessible surface of D437-E438 is highlighted in pink and the buried surface of D303-D304 is highlighted in green. (C) Superimposition of both conserved acidic pairs (Asp303-Asp304 and Asp437-Glu438) of modelled *Vh*GlcNAcase (in magenta stick) with the crystal structure of *Sm*CHB (in green stick) in complex with GlcNAc_2_. N atoms are shown in blue and O atoms in red.

We previously reported cloning and recombinant expression of the gene encoding GH20 *β*-*N*-acetylglucosaminidase from the marine bacterium *V*. *harveyi* [9]. The enzyme, known as *Vh*GlcNAcase (formerly *Vh*Nag2), exhibited exolytic activity, degrading chitin oligosaccharides from the non-reducing end in a sequential manner, with GlcNAc monomer as the final product. Since the crystal structure of *Vh*GlcNAcase is undetermined, we first gained preliminary information on the structural identity of *Vh*GlcNAcase by aligning its sequence with those of other GH20 GlcNAcases of known structure. The results showed that the highest sequence identity of *Vh*GlcNAcase was with *Serratia marcescens* chitobiase (*Sm*CHB), with 24% identity [32], followed by *Streptomyces plicatus β*-*N*-acetylhexosaminidase (*Sp*Hex) with 21% identity [17], human *β*-hexosaminidase A (*Hs*HexA) [33] and human *β*-hexosaminidase B (*Hs*HexB) [10] with 17% identity, while the lowest was with *Paenibacillus* sp. *β*-hexosaminidase (*Ps*Hex1T) which had 13% identity [34]. Structure-based alignment of *Vh*GlcNAcase and *Sm*CHB (Fig 1A) indicated two separate conserved segments on the surface of the (β/α)_8_ TIM barrel domain of the two enzymes. For *Vh*GlcNAcase, the preceding segment comprises the acidic pair Asp303-Asp304, located at the end of loop2 (L2), which links strand β2 and helix α2 (Fig 1A, upper sequence portion). The second pair, Asp437-Glu438, is present at the start of loop4 (L4) connecting strand β4 and helix α4 (Fig 1A, lower sequence portion). Superimposition of the modelled structure of *Vh*GlcNAcase with the crystal structure of *Sm*CHB gave an R.M.S.D. of 0.651 Å for 390 C_α_ atoms (Fig 1B) and showed both conserved acidic pairs to be part of the GlcNAc-binding pocket. The Asp437-Glu438 pair was located close to the scissile bond joining -1GlcNAc and +1GlcNAc, which suggested that these amino acids could play a catalytic role. Structural alignment of the active site residues (Fig 1C) showed that the location of the Asp303-Asp304 pair is equivalent to that of Asp378-Asp379 in *Sm*CHB, whereas the Asp437-Glu438 pair corresponded with Asp539-Glu540 [35]. Based on the crystal structure and kinetic data, the Asp539-Glu540 pair had been suggested to have a catalytic function for *Sm*CHB [35].

### Effects of the active-site mutation on the specific activity of *Vh*GlcNAcase

Point mutations of the selected residues (Asp303, Asp304, Asp 437 and Glu438) caused a drastic loss of enzymatic activity (Table 2, column 2). The activity of mutants D303A, D304A and D437N was undetectable, while the residual activity of other mutants, including D303N, D304N, D437A and E438A/Q, was less than 5% of that of WT *Vh*GlcNAcase (Table 2, column 2). To study the rescue of activity by external nucleophiles, sodium azide and sodium formate, along with other sodium salts (for the chemical structures of azide and formate ions, see Fig A in S1 File), were first tested for their physicochemical effects on the activity of *Vh*GlcNAcase WT. We recently observed that the enzymic activity of the unmutated (wild-type) *Vh*GlcNAcase was inhibited by various sodium salts, including azide, nitrate, formate and chloride [36]. Here, we confirmed their inhibitory effects on the WT activity. As shown in Fig B in S1 File, the specific activity of *Vh*GlcNAcase WT decreased greatly when sodium azide or sodium nitrate was included in the assay medium. On the other hand, sodium formate and sodium chloride showed only moderate effects. For all the ions tested, the degree of inhibition increased with increasing concentration.

**Table 2:** Specific activity of wild-type *Vh*GlcNAcase and its mutants with *p*NP-GlcNAc as substrate in the absence and presence of 1 M sodium salts. The presented values are Mean ± S.D. obtained from experiments carried out in triplicate.

GlcNAcase mutant	Specific activity (nmol/min/μg)		
	No sodium salt	1 M NaN_3_	1 M HCOONa
Wild-type	19.4 ± 0.22 (100)^a^	0.89 ± 0.14 (5)	15.4 ± 0.29 (80)
D303A	n.d.	n.d.	n.d.
D303N	0.81 ± 0.02 (100)	0.31 ± 0.02 (39)	0.75 ± 0.01 (93)
D304A	n.d.	n.d.	n.d.
D304N	0.29 ± 0.01 (100)	0.10 ± 0.01 (35)	0.27 ± 0.02 (92)
D437A	0.05 ± 0.01 (100)	0.01 ± 0.01 (27)	0.09 ± 0.01 (182)
D437N	n.d.	n.d.	n.d.
E438A	0.07 ± 0.01 (100)	0.03 ± 0.01 (46)	0.08 ± 0.01 (102)
E438Q	0.09 ± 0.01 (100)	0.06 ± 0.01 (65)	0.10 ± 0.01 (108)

^a^ Numbers in brackets indicate the relative specific activities of *Vh*GlcNAcase and its variants with each sodium salt, in comparison with *Vh*GlcNAcase without added sodium salt (set to 100).

n.d: undetectable activity.

Next we tested two selected compounds, sodium azide and sodium formate, for their ability to rescue the enzymic activity of the inactive mutants. The results clearly showed that sodium formate had much less inhibitory effect on the *Vh*GlcNAcase mutants than on the WT enzyme (Table 2, column 3). When 1 M sodium azide was included in the assay medium the specific activity of the *Vh*GlcNAcase WT was less than 5% of the original activity, while the residual activity of the mutants was 27–65% of the original. On addition of sodium formate (Table 2, column 4), relatively less inhibition, or even enhancement of activity, was also observed with the enzyme variants. Notably, the specific activity of the D437A mutant was enhanced to 182% of the basal activity in the presence this compound.

### Effects of sodium formate concentration on the rescued activity of the D437A mutant

**Fig 2:**
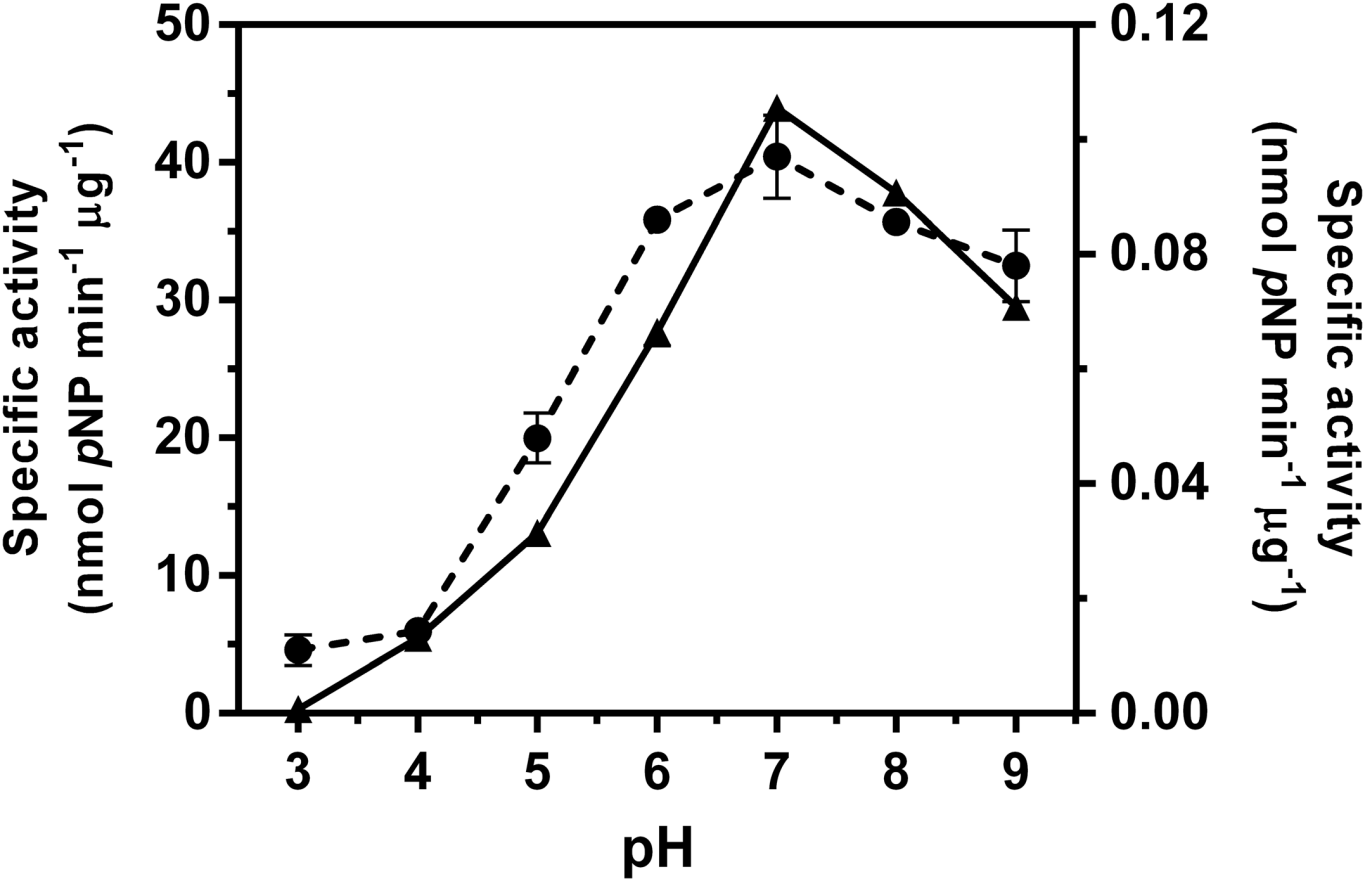
Activity/pH profiles of *Vh*GlcNAcase and its mutant D437A. The specific activity of *Vh*GlcNAcase (solid line, left y axis) and the mutant D437A (dashed line, right y axis) was measured at pH = 3.0, 4.0, 5.0, 6.0, 7.0, 8.0 and 9.0 in the McIlvaine’s sodium phosphate-citric acid buffer system. *p*NP-GlcNAc was used as substrate and the reaction was carried out for 10 min at 37°C.

**Fig 3:**
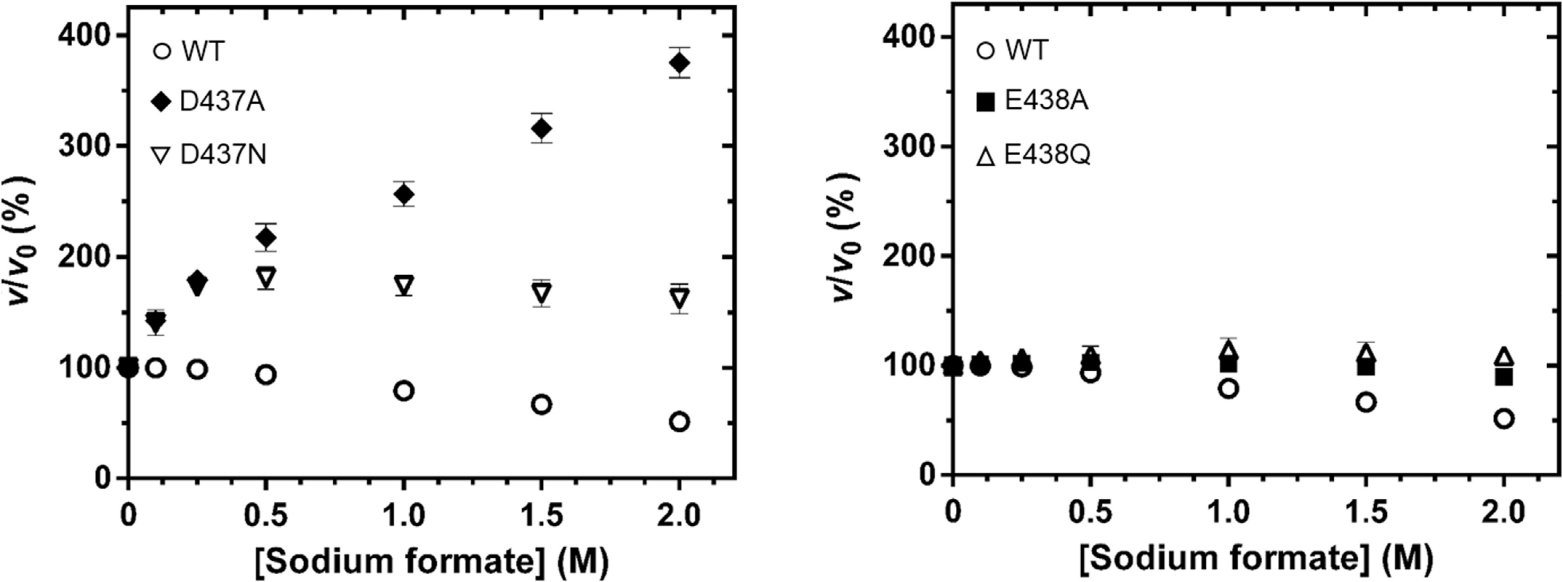
Effect of sodium formate on *p*NP-GlcNAc hydrolysis by *Vh*GlcNAcase and its mutants. Various concentrations of sodium formate (0.0–2.0 M) were added to the reaction mixture, which contained 500 μM *p*NP-GlcNAc and 100 mM sodium phosphate buffer, pH 7.0, at 37°C. *v*/*v*_0_ is the fractional activity of the enzyme, i.e. activity in the presence of sodium formate relative to that in its absence. (A) The D437A (filled diamonds) and D437N (open inverted triangles) mutants. (B) The mutants E438A (filled squares) and E438Q (open triangles). The wild-type *Vh*GlcNAcase (open circles) are shown in both A and B.

Since only for mutant D437A was the specific activity significantly enhanced by sodium formate, we examined whether this mutant showed a shift in the activity/pH curve compared to the WT enzyme. Fig 2 shows the similar response of the activity of the two *Vh*GlcNAcase forms to pH variation. Although mutant D437A had a slightly broader activity/pH curve than the WT enzyme, the two forms had a similar optimal pH of around 7.0. Next, we investigated whether the enzyme activity of the *Vh*GlcNAcase D437A was modified by sodium formate in a concentration-dependent manner. In this set of experiments, we also included the effect of concentration on the activity of the E438A mutant, for comparison. Fig 3 shows plots of the fractional activity (*v*_i/_*v*_0_) of the enzyme at discrete concentrations of sodium formate. The relative activity of *Vh*GlcNAcase WT was found to decline in response to increasing concentrations of sodium formate from 0.1 to 2.0 M; at the highest concentration, the residual activity of the WT enzyme was reduced to less than half of its original value. In marked contrast, the relative activity of mutant D437A was elevated with increasing sodium formate concentration, and at 2.0 M sodium formate was four times the original activity, while the relative activity of mutant D437N increased slightly (about 1.7-fold) (Fig 3A). However an increase in concentration of sodium formate did not restore the enzymatic activity of the E438A or E438Q mutants (Fig 3B). When the same set of enzyme variants was tested with sodium azide, decreases in the fractional activity were observed (data not shown).

### Steady state kinetics of activation by sodium formate

**Fig 4:**
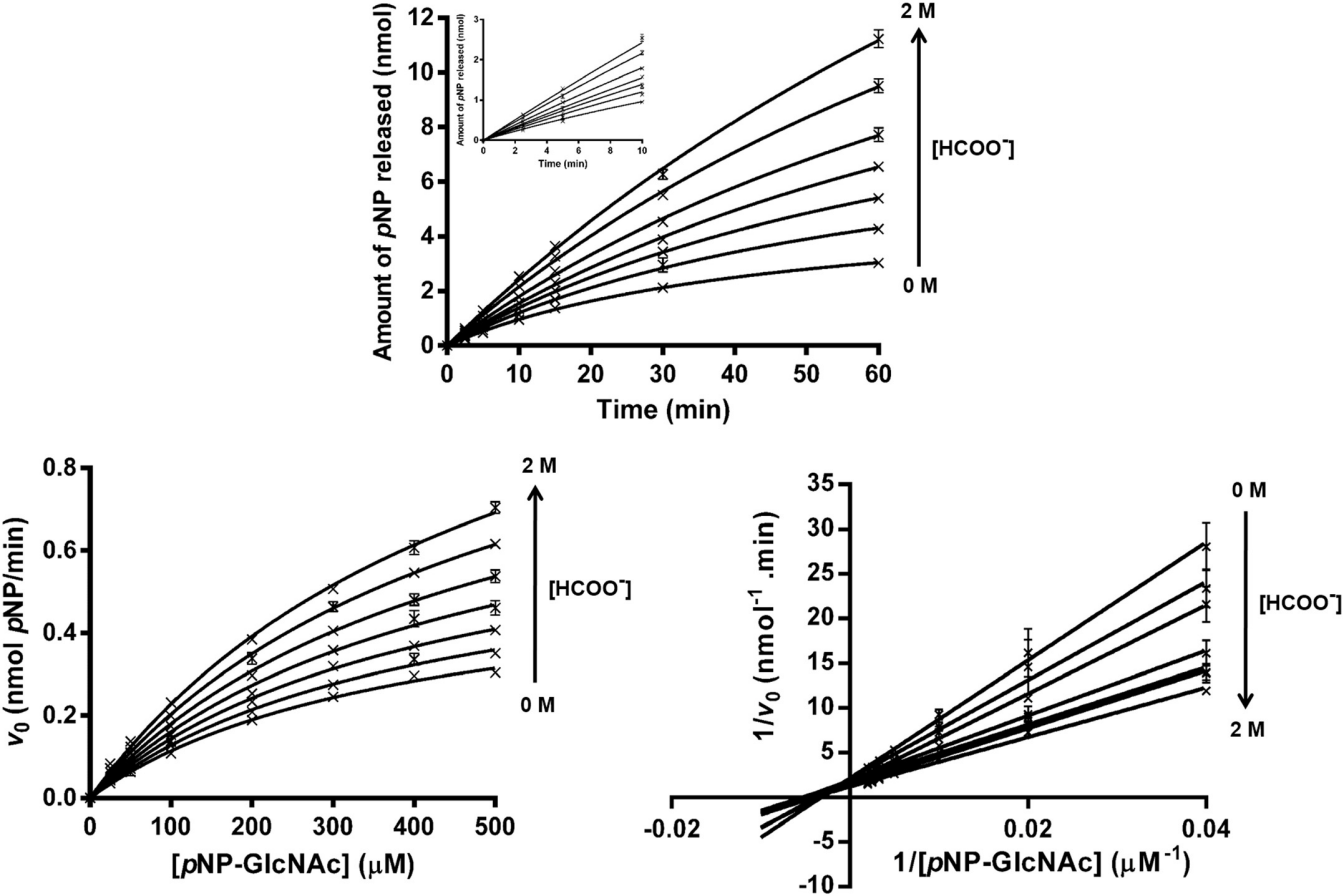
(A) Time-courses of reactions of the D437A mutant with and without sodium formate. Reaction mixtures (200 μL), containing 2 μg of D437A mutant and 500 μM of *p*NP-GlcNAc and varied concentrations of sodium formate (0, 0.1, 0.25, 0.5, 1.0, and 2.0 M) and 100 mM sodium phosphate buffer, pH 7.0, were incubated at 37°C for 0–60 min, and the reaction terminated with 100 μL of 3 M Na_2_CO_3_. Release of *p*NP, monitored at A_405_, was converted to molar quantities using a calibration curve of *p*NP (0–20 nmol). The linear part of the reaction progress was shown as an inset. (B) Initial reaction rates for the mutant D437A of *Vh*GlcNAcase in the presence of sodium formate were obtained from Michaelis-Menten plots. Reaction rates were measured using *p*NP-GlcNAc (0–500 μM) as the substrate, 5 μg of the mutant D437A of *Vh*GlcNAcase and sodium formate at the same range of concentraitons as described above. (C) Activation by formate anion was evaluated by means of Lineweaver-Burk plots of initial reaction rates.

**Fig 5:**
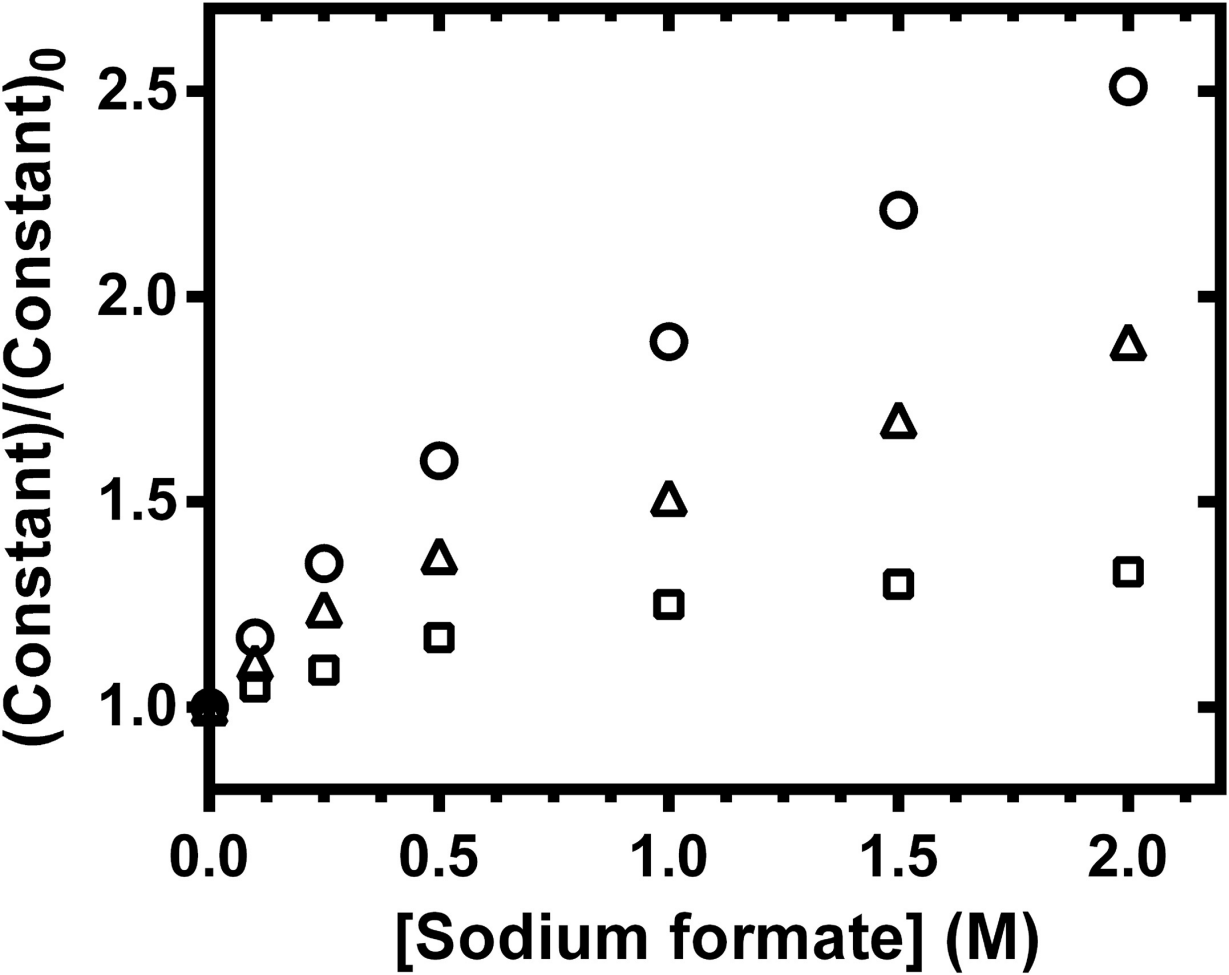
Chemical recue of the D437A mutant by sodium formate. Three kinetic constant ratios: the apparent first-order rate constants, *k*_cat_ (open circles), apparent Michaelis constants, *K*_m_ (open squares) and apparent second-order rate constants, *k*_cat_*/K*_m_ (open triangles), were plotted as a function of sodium formate concentration.

In order to determine the initial rate of reaction, the product generated in the course of *p*NP-GlcNAc hydrolysis by the mutant D437A was monitored at different time points. Fig 4A is a plot of *p*NP release against time, showing that the initial rate of the reaction with and without sodium formate could be determined within 10 min, the amount of product formed up to this time being directly proportional to the time of incubation (Fig 4A, inset). Fig 4B shows non-linear increases in the initial reaction rates for the D437A mutant with increasing substrate concentration, and discrete increases in sodium formate concentrations from 0.1 to 2.0 M. These plots exhibit typical Michaelis-Menten kinetics, where the apparent maximum rate of reaction (app *V*_max_) is approached at concentrations of *p*NP-GlcNAc above 500 μM. Inverse transformation of the non-linear plots in Fig 4B yields linear Lineweaver-Burk (LB) plots, as shown in Fig 4C. Each plot, representing the relation of 1/*v*_0_ and 1/[S], allows estimation of the kinetic parameters of the enzyme in the absence and presence of sodium formate. The kinetic data for the mutant D437A in Table 3 indicate discrete increases in apparent values of *K*_m_, *k*_cat_, and *k*_cat/_*K*_m_. The enhancement of the enzymic activity of *Vh*GlcNAcase on addition of sodium formate is shown in Fig 5 as plots of (app *K*_m_)/*K*_m_, (app *k*_cat_)/*k*_cat_, and (app *k*_cat_/*K*_m_)/(*k*_cat_/*k*_m_), all relative to the values in the absence of formate (constant/constant_0_), as a function of formate concentration. The data analysis indicates a small, concentration-dependent increase in the *K*_m_ value, reaching 1.3-times the reference value at 2.0 M sodium formate. In contrast, very significant increases in the constant *k*_cat_ were observed, and at 2.0 M sodium formate, *k*_cat_ was 2.5 fold greater than at 0 M. Hence, the ratio *k*_cat_/*K*_m_ ration was increased to 1.9-times the reference ratio, in the presence of 2.0 M sodium formate.

**Table 3:** Kinetic parameters for the hydrolytic activity of *Vh*GlcNAcase mutant D437A, in the presence of increasing sodium formate (HCOONa) concentration. The presented values are Mean ± S.D. obtained from experiments carried out in triplicate.

Sodium formate concentration (M)	*K*_m_ (μM)	*k*_cat_ (s^-1^)	*k*_cat_/*K*_m_ (s^-1^ mM^-1^)
0	390 ± 32 (100)^a^	0.14 ± 0.01 (100)	0.36 (100)
0.10	411 ± 37 (105)	0.16 ± 0.01 (117)	0.40 (111)
0.25	425 ± 29 (109)	0.19 ± 0.007 (135)	0.44 (124)
0.50	456 ± 56 (117)	0.22 ± 0.02 (160)	0.49 (137)
1.00	489 ± 41 (125)	0.26 ± 0.01 (189)	0.54 (151)
1.50	509 ± 33 (130)	0.31 ± 0.01 (221)	0.60 (170)
2.00	519 ± 38 (133)	0.35 ± 0.02 (251)	0.67 (189)

^a^ Values in brackets represent relative activity compared to that without sodium formate (set as 100)

## Discussion

Chitin turnover in the marine biosphere depends upon the activities of marine *Vibrios* [37,38]. The chitin catabolic cascade of the *Vibrios* has been demonstrated to involve a large number of genes and enzymes, which are orchestrated in a complex signal transduction pathway [2,3,37–41]. We previously identified and characterized three biological components of the chitin catabolic pathway that are essential for chitin degradation and chitin uptake by *V*. *harveyi*. Chitinase A (so-called *Vh*ChiA) is an endolytic enzyme responsible for the breakdown of insoluble chitin chains into small, soluble chitooligosaccharides [8,42], while chitoporin (so-called *Vh*ChiP), a sugar-specific porin located in the outer membrane of the bacterium, is responsible for chitooligosaccharide uptake [6,7]. The last component is *β*-*N*-acetylglucosaminidase (known as *Vh*GlcNAcase or formerly *Vh*Nag2), an exolytic enzyme capable of degrading the transported chitooligosaccharides to GlcNAc monomers, which then act as signalling molecules that regulate the downstream cascade of the chitin catabolic pathway, through the activation of the chitin sensor (ChiS) [40,43]. *Vh*GlcNAcase, a member of the GH20 GlcNAcase family, contains four GlcNAc binding subsites (-1), (+1), (+2) and (+3), and exhibits its greatest activity with chitotetraose [9]. Amino acid sequence comparison with other GlcNAcases and our 3D-structure, modelled on the known 3D structure of *Sm*CHB (Fig 1A, 1B and 1C), suggested that two invariant acidic side-chain pairs, Asp303-Asp304 and Asp437-Glu438, could be important for catalysis. Both acidic pairs lie in close proximity to the cleavage site (-1 subsite) and have equal opportunity to act as the catalytic couple. In this study, we performed site-directed mutagenesis, followed by a chemical rescue assay, to identify the catalytic couple. In the first set of experiments, we observed that point mutations of four invariant acidic residues (Asp303, Asp304, Asp437 and Glu438) caused a drastic loss of the enzymic activity of *Vh*GlcNAcase toward a synthetic substrate, *p*NP-GlcNAc. Notably, mutations of Asp437 to Ala (mutant D437A) and of Glu438 to Ala (mutant E438A) abolished the activity almost completely, confirming that these acidic residues play important roles in chitin degradation.

In the next experiment, we observed that among various sodium salts, sodium azide greatly inhibited the activity of *Vh*GlcNAcase WT, but sodium formate produced only weak inhibition (Fig B in S1 File). Such observations were consistent with our previous report that sodium azide acted as a potent competitive inhibitor of *Vh*GlcNAcase [36]. Both azide and formate ions, the forms of sodium azide and sodium formate, respectively, that exist in buffered solution, are strong nucleophiles [44,45]. Therefore, their ability to rescue enzymic activity of inactive mutants through nucleophilic effect has been employed to elucidate the catalytic mechanism of several retaining glycoside hydrolases [19,23–27]. In our study, their inhibitory effects on the mutant forms of *Vh*GlcNAcase were significantly less than on WT, suggesting that the inactivating effects of the active-site mutations were partially eliminated when azide or formate was included in the assayed reaction. Formate ion appeared to act as the more potent nucleophile, as we observed its greater chemical rescue effect on the mutants D437A and E438A/Q, as compared to that of azide ion; the enzymic activity of the mutant D437A was even enhanced by formate, but not by azide. The less effective chemical rescue produced by azide ion may result from its linear geometry, which allows only a poor fit into the catalytic pocket of *Vh*GlcNAcase. In contrast formate ion, which has trigonal planar geometry, may accurately mimic the carboxylate side chain of Asp437 (Fig A in S1 File). Therefore, the activity loss due to the interruption of the catalytic cycle, caused by loss of the natural nucleophile upon replacement of Asp437 with Ala, could be re-established in the presence of this small exogenous nucleophile.

**Fig 6:**
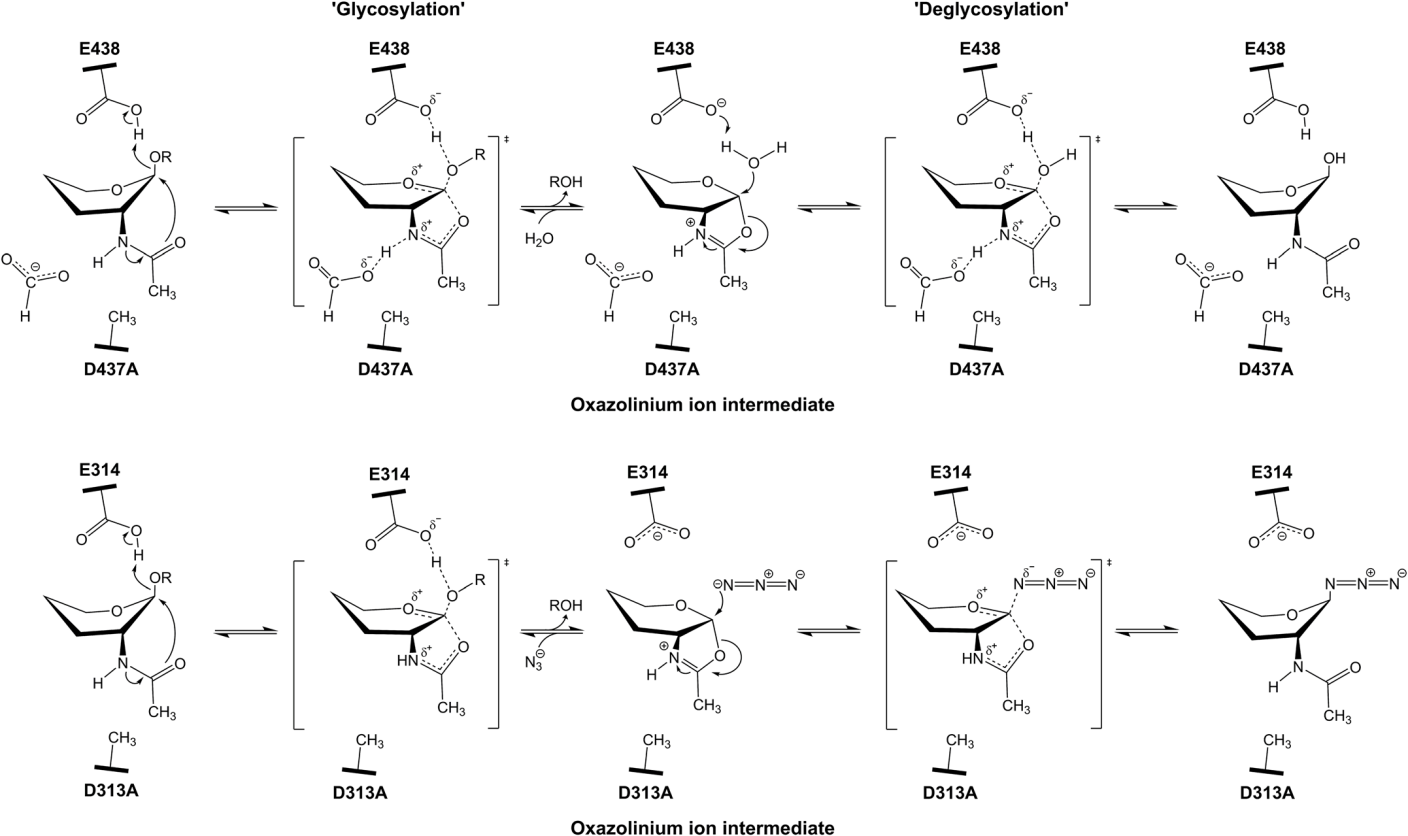
(A) Proposed mechanism of formate-mediated chemical rescue of the activity of the *Vh*GlcNAcase D437A mutant. Formate ion is involved in both the glycosylation and deglycosylation steps by providing charge stabilization of transition states that flank the oxazolinium ion. (B) Proposed mechanism of azide-mediated chemical rescue with the *Sp*Hex D313A mutant. Azide ion is involved only in the deglycosylation step, acting to open the cyclic oxazolinium ion intermediate [21]. Hydroxyl groups and C_6_ have been omitted for clarity.

Lineweaver Burk plots of 1/*v*_o_ vs. 1/[s] at different formate concentrations (Fig 4C) yielded lines that intersect above the x-axis, agreeing with the mix-type mode of binding. The results suggested that formate ion could interact with both unliganded D437A (E^mut^) and ligand-bound D437A (E^mut^S), but the enhanced activity would occur only when formate ion bound to the enzyme-substrate complex. Fig 6 shows the proposed mechanism, in which formate ion increases the rate of *p*NP-glycoside hydrolysis by replacing the substituted side-chain of Asp437 in the catalytic pocket of the D437A-substrate complex. The restoration of the enzyme activity observed with the mutant D437A suggested a crucial role for Asp437 as the catalytic nucleophile in both the glycosylation and deglycosylation steps of the substrate-assisted mechanism proposed for GH20 GlcNAcases [21].

The absence of any shift in the optimal pH in the pH-activity profiles of the *Vh*GlcNAcase WT and the D437A mutant suggested that D437 did not facilitate bond cleavage by lowering the p*K*_a_ value of its catalytic partner Glu438. This is a major difference in the catalytic role of Asp437 in *Vh*GlcNAcase from Asp313 in *Sp*HEX. In the case of *Sp*HEX, the D313A mutant was shown to have its optimal pH value increased from 5.0 to 7.5, the pH/activity data suggesting a significant contribution to bond cleavage by Asp313 [21].

When compared with *Sp*HEX [21], we propose that the effects of formate ion on the *Vh*GlcNAcase inactive mutant are mechanistically different from the effects of azide ion on the *Sp*HEX inactive mutant. As shown in Fig 6A, formate ion acts as a nucleophilic substitute for Asp437 in the *Vh*GlcNAcase D437A mutant, the planar formate ion (HCOO^-^) optimally filling the volume occupied in WT *Vh*GlcNAcase by the β-COO^-^ side chain. Formate then accepts a proton from the -NH of the C2-acetamido group of the oxazolinium intermediate that is generated in course of the scissile-bond cleavage by the acid catalyst Glu438. Such covalent bond formation aids the stabilization of the oxazolinium intermediate in the glycosylation step, and also helps to orient the positively charged C1 of the reaction intermediate, so as to react with the neighbouring water molecule in the subsequent deglycosylation step. Our data show that formate-mediated chemical rescue produced similar increases in (apparent *k*_cat_)/*k*_cat_, and (apparent *k*_cat_/*K*_m_)/(*k*_cat_/*k*_m_) (2.5- and 1.9-fold respectively), supporting the catalytic role of Asp437 in both glycosylation and deglycosylation steps in the enzyme-substrate complex. In marked contrast, azide ion rescued the GlcNAcase activity of *Sp*HEX inactive mutant (D313A) by acting as an alternative nucleophile to water (not to Asp313), generation a β-glycosyl azide product. As shown in Fig 6B, azide ion does not mimic the nucleophilic role of Asp313, but the result of the nucleophilic attack by azide ion is to open the oxazolinium ion intermediate during the deglycosylation step. This proposed mechanism was revealed by the kinetic analysis, which showed a much greater increase in the first-order rate constant ratio (apparent *k*_cat_)/*k*_cat_ (16-fold) than in the second-order rate constant ratio (apparent *k*_cat_/*K*_m_)/(*k*_cat_/*k*_m_) (5-fold). In *Vh*GlcNAcase such analysis suggested that the exogenous nucleophile restored the activity of the D437A mutant by accelerating both the rate of deglycosylation (as reflected by the apparent *k*_cat_) and of glycosylation (as reflected by the apparent *k*_cat_*/K*_m_). It is noteworthy that the rescue effect observed for our inactive *Vh*GlcNAcase was not dramatic, and this may reflect the reactivity of the leaving group on the tested substrate. *p*NPGlcNAc contains a poor leaving group, so is much less susceptible to enzymic hydrolysis than 2,4DNPGlcNAc and 3,5DNPGlcNAc, which contain strong leaving groups. Similar results were reported by Vallmitjana et al. [23]. They observed only a 3 fold enhancement of *k*_cat_ when *p*NPG was used as the substrate for the *β*-glucosidase assay of the nucleophilic inactive mutant E178A, while 188 fold *k*_cat_ enhancement was observed when 2,4DNPG was the substrate. This would explain the modest 2.5 fold increase in *k*_cat_ for *Vh*GlcNAcase inactive mutant with *p*NPGlcNAc as substrate, compared to *Sp*HEX, which showed a 16 fold increase in *k*_cat_ with 3,5DNPGlcNAc substrate [21].

## Conclusions

In this study, we have demonstrated that an exogenous nucleophile (formate ion) selectively enhances the enzymatic activity of an inactive mutant *Vh*GlcNAcase, D437A, in a concentration-dependent manner. However, the activity of other active-site mutants (D303A/N, D304A/N, and E438A/Q) was not significantly affected by the addition of this strong nucleophile. The rescued activity of the D437A mutant suggests that Asp437 is the catalytic nucleophile, while its invariant acidic partner Glu438 likely acts as a catalytic proton-donating residue. This experimental evidence confirms that the residues Asp437 and Glu438, located in the middle of the substrate-binding cleft in the modelled structure of GH20 *Vh*GlcNAcase, act as the catalytic pair in the catalytic cycle of chitooligosaccharide hydrolysis by this enzyme.

## Supporting Information

S1 File The chemical structures of azide and formate ions used in this study (Fig A) and specific hydrolytic activity of wild-type *Vh*GlcNAcase against pNP-GlcNAc, in the presence of various concentrations of sodium salts (Fig B). (PDF)

## Abbreviations

*Vh*GlcNAcase: *Vibrio harveyi β*-*N*-acetylglucosaminidase
GH: glycoside hydrolase
GlcNAc: *N*-acetylglucosamine
*p*NP-GlcNAc: *para*-nitrophenyl-*N*-acetylglucosaminide
IPTG: isopropyl *β*-D-1-thiogalactopyranoside
PMSF: phenylmethylsulfonyl fluoride
