# Supplementary figures and images for: Probing the Catalytic Mechanism of Vibrio harveyi GH20 β-N-Acetylglucosaminidase by Chemical Rescue
Source: PLoS One. 2016 Feb 12;11(2):e0149228. doi: 10.1371/journal.pone.0149228 (PMC4752478; doi:10.1371/journal.pone.0149228)

**Fig. A**

**Azide anion ( $\text{N}_3^-$ )**

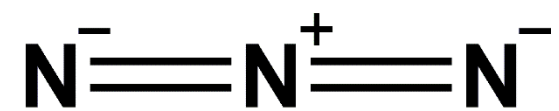

**Formate anion ( $\text{HCOO}^-$ )**

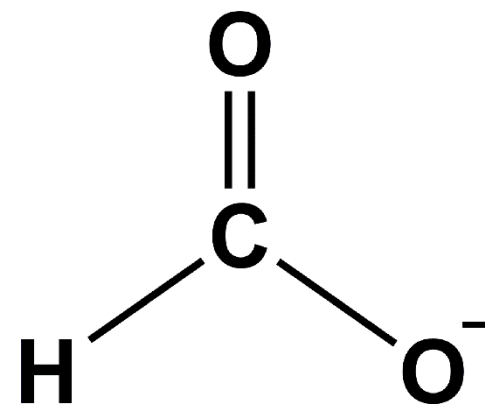

**Fig. B**

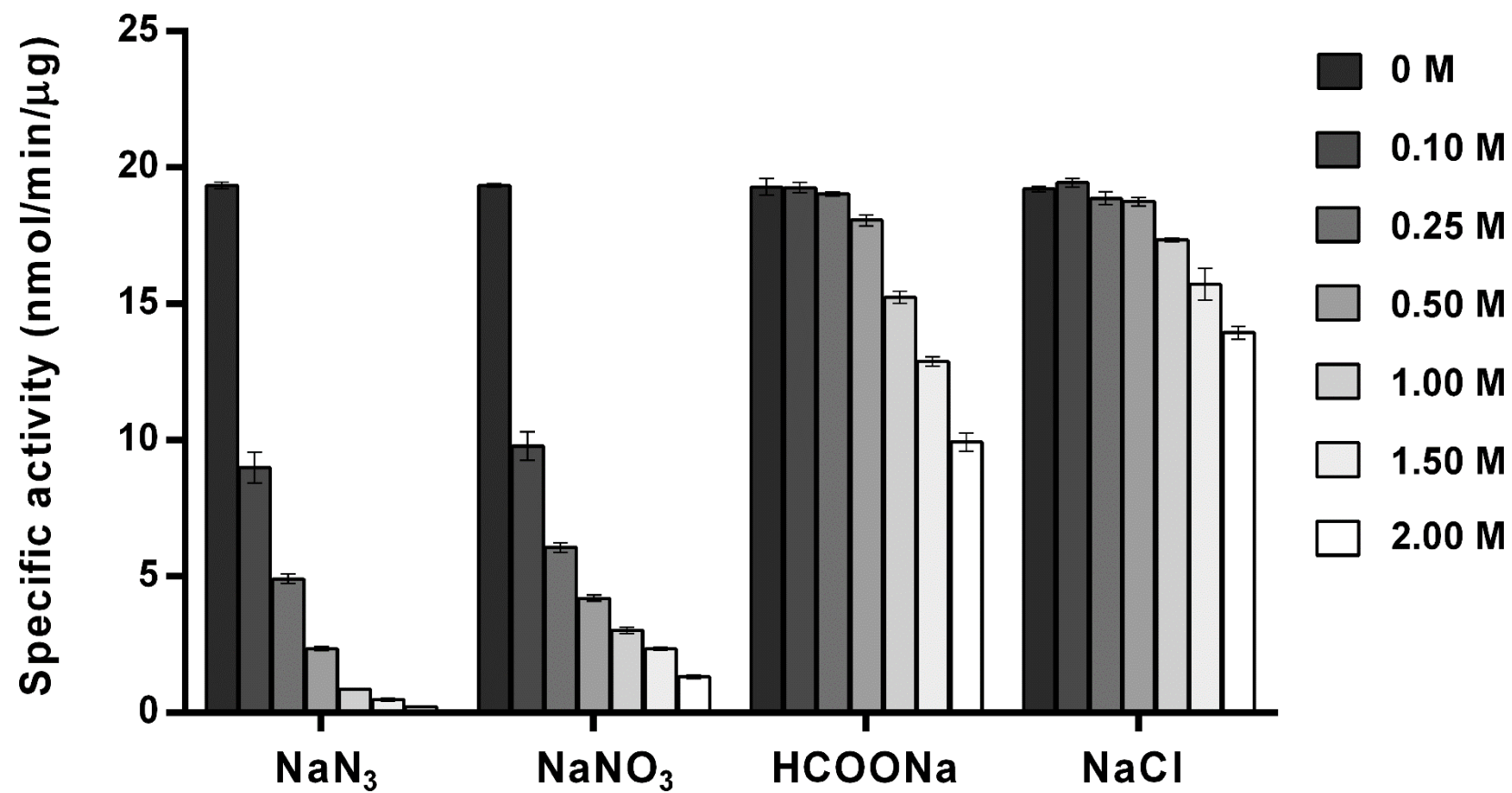

Supplement: S1 File — (PDF) [file pone.0149228.s001.pdf]
